# Development of a Database of Health Insurance Claims: Standardization of Disease Classifications and Anonymous Record Linkage

**DOI:** 10.2188/jea.JE20090066

**Published:** 2010-09-05

**Authors:** Shinya Kimura, Toshihiko Sato, Shunya Ikeda, Mitsuhiko Noda, Takeo Nakayama

**Affiliations:** 1 Japan Medical Data Center Co, Ltd, Tokyo, Japan; 2 Kitasato Clinical Research Center, Kitasato University School of Medicine, Kanagawa, Japan; 3 Department of Pharmaceutical Sciences, School of Pharmacy, International University of Health and Welfare, Tochigi, Japan; 4 Department of Diabetes and Metabolic Medicine, National Center for Global Health and Medicine, Tokyo, Japan; 5 Department of Health Informatics, Kyoto University School of Public Health, Kyoto, Japan

**Keywords:** database, administrative data, health insurance claims, record linkage, health services research

## Abstract

**Background:**

Health insurance claims (ie, receipts) record patient health care treatments and expenses and, although created for the health care payment system, are potentially useful for research. Combining different types of receipts generated for the same patient would dramatically increase the utility of these receipts. However, technical problems, including standardization of disease names and classifications, and anonymous linkage of individual receipts, must be addressed.

**Methods:**

In collaboration with health insurance societies, all information from receipts (inpatient, outpatient, and pharmacy) was collected. To standardize disease names and classifications, we developed a computer-aided post-entry standardization method using a disease name dictionary based on International Classification of Diseases (ICD)-10 classifications. We also developed an anonymous linkage system by using an encryption code generated from a combination of hash values and stream ciphers. Using different sets of the original data (data set 1: insurance certificate number, name, and sex; data set 2: insurance certificate number, date of birth, and relationship status), we compared the percentage of successful record matches obtained by using data set 1 to generate key codes with the percentage obtained when both data sets were used.

**Results:**

The dictionary’s automatic conversion of disease names successfully standardized 98.1% of approximately 2 million new receipts entered into the database. The percentage of anonymous matches was higher for the combined data sets (98.0%) than for data set 1 (88.5%).

**Conclusions:**

The use of standardized disease classifications and anonymous record linkage substantially contributed to the construction of a large, chronologically organized database of receipts. This database is expected to aid in epidemiologic and health services research using receipt information.

## INTRODUCTION

Health insurance claims, hereafter referred to as receipts, record patient health care treatments and expenses, and therefore can be utilized to analyze current health care conditions in Japan.^1^^–^^5^ Several types of receipts exist, including those for medical, dental, pharmacy, visiting nursing care, and bone setting services. Each medical institution, pharmacy, and health care agency issues a receipt every month for each patient. The Social Insurance Medical Fee Payment Fund (MFPF) and the All-Japan Federation of National Health Insurance Organizations issue approximately 70 million and 50 million receipts per month, respectively.^6^^,^^7^ The total number of receipts exceeds 1 billion per year in Japan. Each receipt lists the patient’s name, date of birth, and insurance certificate number, the medical institution’s name, the disease name, health care expenses, and services received. Insurers such as municipalities and health insurance societies use these receipts to make payments to medical institutions and pharmacies. Receipts are first submitted to review organizations like MFPF. After cross-checking the entered data and verifying the claims and health care expenses, receipts are submitted to insurers. To ensure that health care expenses are appropriate, insurers carry out their own review of the receipts and request reexaminations of incorrect claims.

Combining the different types of receipts for each person would dramatically increase the utility of receipts; however, the following issues have been identified:

(1) The disease name recorded on receipts varies by health care institution.
(2) Privacy must be protected because receipts contain personal information such as full names.
(3) If the same individual is examined at multiple institutions in the same month, analysis of receipts from individual institutions does not reflect all the individual’s examinations and treatments.

As an example of issue 1 above, type 2 diabetes may be recorded as NIDDM (non-insulin dependent diabetes mellitus), diabetes II, or adult diabetes. The standardized disease-code master is the system used for standardizing disease names in Japan, and is developed and maintained by the Medical Information System Development Center (MEDIS-DC) commissioned by the Ministry of Health, Labour and Welfare (MHLW).^8^ This master includes approximately 20 000 disease names, which are compliant with the International Classification of Diseases (ICD-10, version 2003). When creating electronic databases, health information managers at individual medical institutions will often code diseases on the receipts using the ICD-10 classifications, which can lead to several problems: mistakes can lead to the coding of a single disease with different names; manual coding requires compliance with rules and shared knowledge among staff, costing the institution time and money in staff training; once entered, the disease name cannot be updated to reflect periodic revisions in the standard disease code master, introducing errors in time-series analyses; and in some cases, disease names entered onto receipts using the ICD-10 names cannot be subsequently changed back to the standardized disease names. Consequently, a more efficient and reliable method of ICD10-based standardization of disease names and classification is needed.

**Figure 1.**
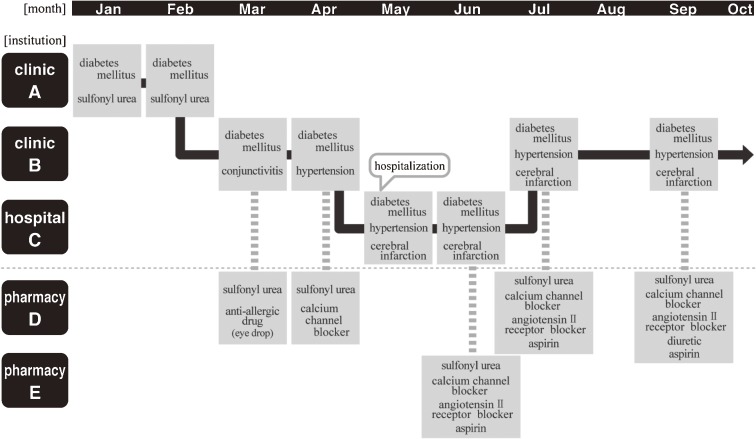
An example of the chronological process of issuing receipts for an individual

Issues 2 and 3 can be regarded as issues related to anonymous linkage of information from multiple receipts. In a given month, a patient may visit a clinic, be admitted to a hospital after clinic referral, return to the clinic after hospital discharge, and receive medication at a pharmacy. As a result, the insurer receives 3 different types of receipt during the month. Figure 1 shows a hypothetical example of such a patient. Name identification (ie, recognizing the patient on multiple receipts as the same individual) links patients with all their conditions, health care services, and medications received. To maintain prospective accumulation of newly issued receipts and link them to the same person, a system that can remove personal information and automatically perform name identification is needed. Tracking an individual’s health care history through multiple receipts will augment the utility of receipts, from their original use for health care payment to use in epidemiologic or health services research. We developed new methods to resolve the technical problems of establishing a large and growing receipts database in Japan.

## METHODS

In August 2003, a database was begun for the purpose of accumulating receipts, under the terms of a contract between the Japan Medical Data Center (JMDC), a health care venture company established in 2001, and several health insurance societies. From these health insurance societies, JMDC obtains all information from receipts issued by each medical institution, collects them on paper and on DVD, and registers them into the database. The paper media are scanned as image data and digitized using an OCR (optical character reader).

### Ensuring reproducibility of disease names and classifications

**Figure 2.**
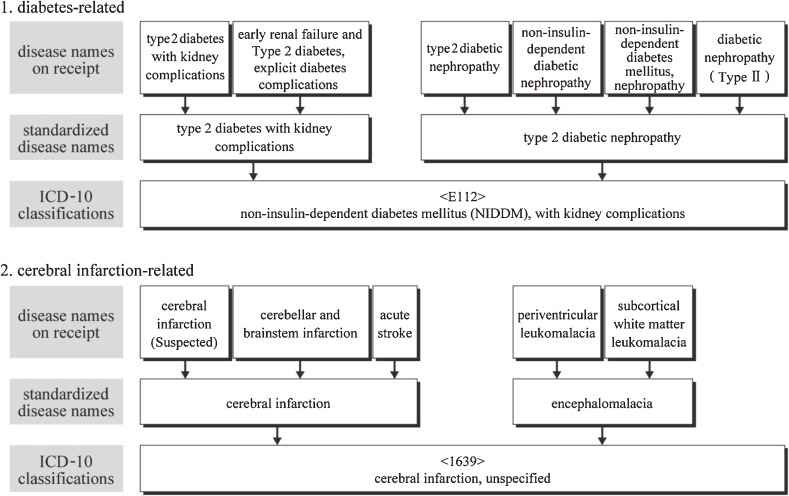
Relationship between receipt disease names, standardized disease names, and ICD-10 classifications

To improve ICD10-based standardization of disease names and classification, we used a computer-aided post-entry standardization method, in which a disease name is entered on receipts in the conventional manner and is standardized after data entry, using a dictionary of disease names. For this purpose, we created a dictionary that links known disease name alternatives to standardized disease names. This master file registered all disease names (13 million names) recorded in approximately 4.8 million medical receipts from 59 110 health care institutions in Japan. Figure 2 shows some examples of this process. The dictionary is updated by monthly receipt processing, resulting in continuous improvements in the percentage of entries that can be automatically standardized.

### Anonymous computer-aided name identification and linkage

**Figure 3.**
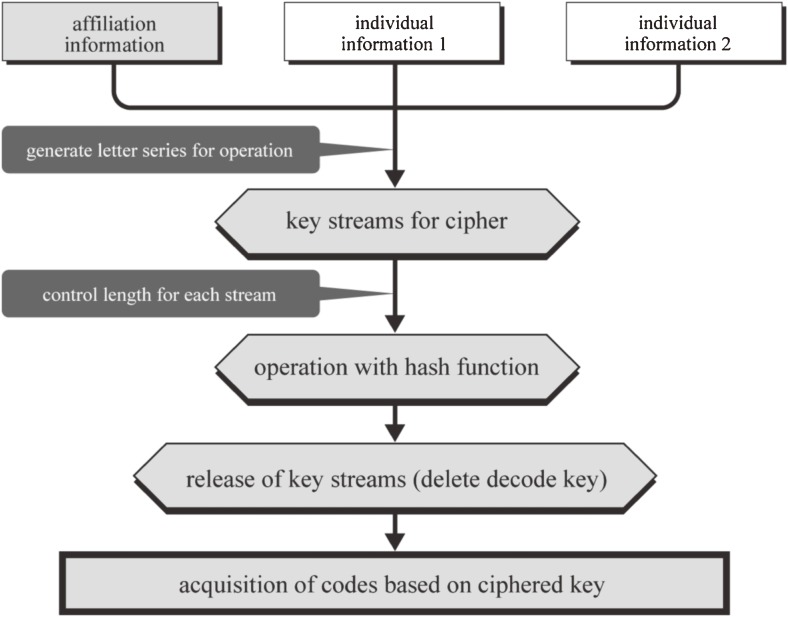
Combined use of a stream cipher and a hash function

To develop the anonymous name identification program, we used an encryption code (key code) generated from the combination of hash values and a stream cipher. A hash function converts a long string of characters, such as a sentence or series of numbers, into certain lengths of data called hash values.^9^^–^^11^ Hash values are the product of irreversible 1-way functions, so the original data cannot be regenerated from hash values. The stream cipher uses a pseudorandom number generator to generate a pseudorandom bit sequence.^12^ When a hash function is used directly for original data, different lengths of letter series (hash values) are generated according to the length of the original data set. To reduce the risk of inferring original data set from the lengths of hash values, the authors first generated the preliminary code by using a stream cipher for the original data, to limit their lengths to within a certain range. Later, a hash function was applied to these preliminary codes (ciphered key) to generate secondary codes. In addition, the decode key that the stream cipher had generated was released (deleted), thereby greatly improving security. Finally, the secondary codes were used as the actual key code for anonymous name identification. Figure 3 shows the process of combining stream cipher and hash functions.

**Figure 4.**
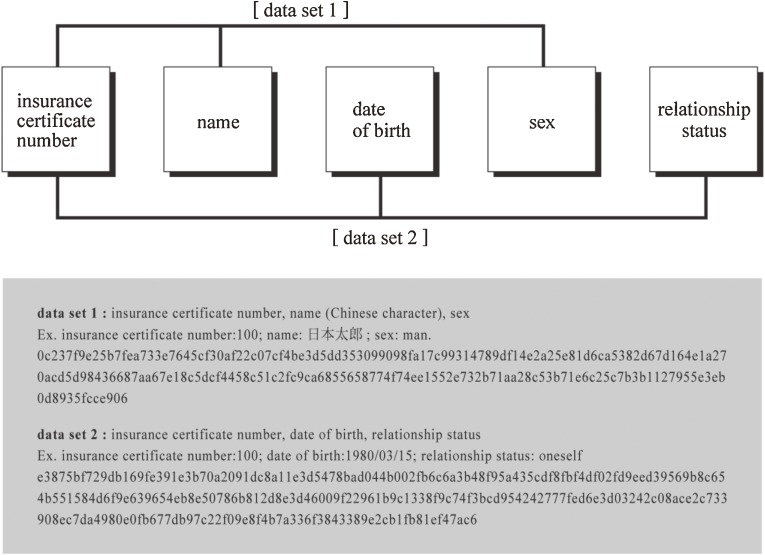
Example of original data used for key code generation in name identification

A key code cannot be generated when the data necessary for generating it are missing from the original information. To improve the success of matching, it may be necessary to utilize more than 1 pattern for generating key codes (ie, use different sets of original data), and to combine them. To examine the validity of this method, the authors compared the success of name identification using data set 1 with a method that combined 2 data sets. For the first method to generate a key code, we generated hash values and a stream cipher from 3 items in the original data—insurance certificate number, name (Chinese characters), and sex (data set 1)—and performed name identification. In the second method, the insurance certificate number, date of birth, and relationship status (data set 2) were used along with data set 1 to generate a key code. The results using data set 1 were then compared with those obtained using the combined data sets (Figure 4).

## RESULTS

Most (70%) of the information from receipts for insured individuals is stored on DVDs; the other 30% represents images scanned from paper. Both these data sources were successfully included in the database.

Using the dictionary for post-entry standardization, approximately 2 million new receipts were processed for disease names and classifications, and 98.1% of these receipts were automatically standardized.

**Figure 5.**
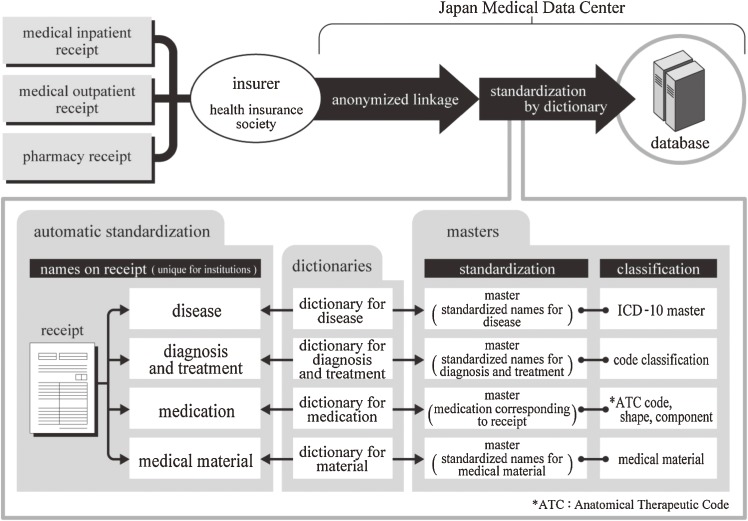
System for developing the receipt database

Regarding anonymous linkage, when name identification was performed for 101 700 medical outpatient receipts, with prescription expenses as a population parameter, the matching percentage for pharmacy receipts was 88.5% using data set 1; however, the percentage improved to 98.0% when name identification was performed using key codes from data sets 1 and 2 combined. Errors in receipt data entry and insurance certificate numbers accounted for the unmatched 2%. Figure 5 summarizes the system that was developed.

## DISCUSSION

The 2 methods reported in this article have substantially aided in the construction of a large database of receipts, and improved the usefulness of such receipts in 2 ways.

First, the database uses a standardized disease classification, so that various disease names and classifications entered at different health care institutions are automatically converted to their standardized forms. The advantages of using post-entry standardization with the present system’s unique dictionary, rather than simply converting disease names to the ICD-10 classification during data entry, are that:

(1) Reproducibility of data is improved, as manual coding is not necessary.
(2) By automatically updating previously entered data, dictionary updates enable accurate time-series analysis.
(3) Converting disease names on receipts to standard disease names generates more information than does direct conversion into ICD-10 names. For example, the cases that are classified under the ICD-10 code, “E142: Unspecified diabetes mellitus with renal complications,” can be recorded and analyzed separately as diabetic nephropathy, diabetic renal failure, Kimmelstiel-Wilson syndrome, and diabetic nephrosclerosis.
(4) The dictionary is able to distinguish between suspected and definite diagnoses.

During standardization of disease names, the post-entry standardization method was unable to process 1.9% of recorded disease names, because they had not yet been registered in the dictionary. The authors plan to improve the performance of the dictionary by gradually adding these disease names.

The second advantage is that a database with anonymously linked information from individual receipts is now available for multiple purposes. Hash functions are often used in bioinformatics^13^^–^^15^; however, their use in health services and epidemiologic research has been limited.^16^ Since 2007, the MHLW has discussed the use of hash functions for anonymous name identification in the launching of the so-called “specific health checkup,” a new system of public health screening for lifestyle-related diseases, particularly metabolic syndrome, for adults aged 40 to 74.^17^ The basic principles of the system used in the present report are similar to those of the MHLW system, but the present system is the first database developed to collect health information such as that included in receipts. Moreover, the present system includes a much more secure system for protecting individual information. When generating encrypted codes, a stream cipher automatically produces the keys for encoding and decoding simultaneously. Then, the people who generate the codes release the decoding key to those who use it. This system is similar to the one used for secure transactions on the internet. In addition to the use of hash functions, the automatic release (deletion) of decode keys produced by the stream ciphers makes the inference of original individual information almost impossible. Concerning anonymous name identification, the authors showed that the combined use of 2 data sets for generating key codes resulted in a better match percentage than the use of a single data set. The authors are now using a more advanced system, in which 27 patterns of combinations of original individual information on receipts are used for generating key codes, resulting in 99.88% success in matches. The unmatched (0.12%) receipts are classified as records from different people. Our system also differs from that used by MHLW because it generates key codes not only from receipt information, but also from information from the insurers’ actual registries of insured people. This is advantageous because a receipt is not issued when insured individuals use no health care service. Thus, these people would be overlooked when only receipts are linked. By using information from insurers’ registries, the present system extends links with other data sources, such as the results of periodic health checkups.

There are a number of differences between traditional epidemiologic studies and analyses using receipt information. Methods for collecting information (exposure/outcome) in traditional epidemiologic studies include obtaining information directly from participants via interviews, questionnaires, and physical or medical examinations, medical records, disease registries, death records, and environmental measurements (atmosphere, noise, toxic substances, etc.). Receipt information is a new source of epidemiologic information.

When using receipt information, its properties and limitations should be kept in mind. One advantage of using receipt information as administrative data is that it is an almost complete collection of information on diagnostic procedures, treatment, and expenses. It is also brings together information on inpatient, outpatient, and in-home medical practices, without being restricted to a single health care institution. However, because multiple disease names are recorded on receipt information, there is a limit to analyzing expenses for individual diseases. Okamoto has attempted to estimate health care expenses for individual diseases from receipt information using the Proportional Disease Magnitude method (PDM).^5^ The present database provides researchers with a sufficient amount of real receipt data to examine the methodological validity of expense estimates for individual diseases.

The other problem regarding the validity of the disease names recorded on receipts is that there are instances where a “suspected disease name” or a “disease name for receipt” are recorded to include fees for reimbursed tests and medications. This leads to an overestimation of patient numbers when measuring disease frequency or extracting patients with that disease. Unfortunately, diseases recorded on receipts may not have been proactively detected using set diagnostic criteria. Thus, information on latent diseases that may be difficult for a physician to diagnose during a routine examination cannot always be obtained from receipts (eg, chronic obstructive pulmonary disease, depression, and insomnia). In addition, test results and information on a patient’s attitude cannot be obtained solely from receipt information. To allow for determination of whether a patient has died, it will be necessary to evaluate the completeness of information on outcomes and supplemental health insurance benefits (such as burial), and information from the insurer’s registry. Moreover, we hope to add receipts for dental care, visiting nursing care, and bone setting to this database.

In summary, we used 2 new methods—standardized disease classification and anonymous record linkage—to construct a large database of receipts organized chronologically. It is expected that this database will promote epidemiologic and health services research that uses receipt information.
